# Enhancing the capacity of community health workers in prevention and control of epidemics and pandemics in Wakiso district, Uganda: evaluation of a pilot project

**DOI:** 10.1186/s12875-024-02522-1

**Published:** 2024-07-17

**Authors:** David Musoke, Grace Biyinzika Lubega, Belinda Twesigye, Betty Nakachwa, Michael Obeng Brown, Linda Gibson

**Affiliations:** 1 Department of Disease Control and Environmental Health, School of Public Health, College of Health Sciences, Makerere University, P. O. Box 7072, Kampala, Uganda; 2 Institute of Health and Allied Professions, School of Social Sciences, Nottingham Trent University, Nottingham, NG1 4FQ UK

**Keywords:** Community health workers, Control, COVID-19, Epidemics, Evaluation, Outbreaks, Pandemics, Prevention

## Abstract

**Background:**

Community Health Workers (CHWs) play a crucial role in outbreak response, including health education, contact tracing, and referral of cases if adequately trained. A pilot project recently trained 766 CHWs in Wakiso district Uganda on epidemic and pandemic preparedness and response including COVID-19. This evaluation was carried out to generate evidence on the outcomes of the project that can inform preparations for future outbreaks in the country.

**Methods:**

This was a qualitative evaluation carried out one year after the project. It used three data collection methods: 30 in-depth interviews among trained CHWs; 15 focus group discussions among community members served by CHWs; and 11 key informant interviews among community health stakeholders. The data was analysed using a thematic approach in NVivo (version 12).

**Results:**

Findings from the study are presented under four themes. (1) Improved knowledge and skills on managing epidemics and pandemics. CHWs distinguished between the two terminologies and correctly identified the signs and symptoms of associated diseases. CHWs reported improved communication, treatment of illnesses, and report writing skills which were of great importance including for managing COVID-19 patients. (2) Enhanced attitudes towards managing epidemics and pandemics as CHWs showed dedication to their work and more confidence when performing tasks specifically health education on prevention measures for COVID-19. (3) Improved health practices such as hand washing, vaccination uptake, and wearing of masks in the community and amongst CHWs. (4) Enhanced performance in managing epidemics and pandemics which resulted in increased work efficiency of CHWs. CHWs were able to carry out community mobilization through door-to-door household visits and talks on community radios as part of the COVID-19 response. CHWs were also able to prioritize health services for the elderly, and support the management of patients with chronic diseases such as HIV, TB and diabetes by delivering their drugs.

**Conclusions:**

These findings demonstrate that CHWs can support epidemic and pandemic response when their capacity is enhanced. There is need to invest in routine training of CHWs to contribute to outbreak preparedness and response.

**Supplementary Information:**

The online version contains supplementary material available at 10.1186/s12875-024-02522-1.

## Introduction

Uganda has been affected by several epidemics in recent years including Ebola, Marburg, Rift Valley Fever, Measles, Cholera, Crimean Congo Hemorrhagic Fever, Anthrax, and Meningitis [1, 2]. These epidemics have led to increased morbidity and mortality, as well as impacted on the broader health system including service delivery, human resources, and access to essential medicines [3]. The country has also been negatively affected by the COVID-19 pandemic which resulted in severe health and social impacts. Uganda experienced 3 main waves of the COVID-19 pandemic, with the second one in 2021 being the most severe [4]. This wave was characterised by widespread community transmission, increased hospital admissions, and high mortality among many age groups particularly the elderly [5].

The pandemic greatly affected health services in Uganda and as such, the number of maternal health complications increased during lockdown [6, 7]. In addition, child health indicators such as immunisation attendance declined, while neonatal deaths increased [6, 7]. Many studies conducted in Uganda have also reported reduced availability and access to chronic care services for HIV/AIDS and non-communicable diseases during the pandemic [8, 9]. There is also evidence of increased mental health conditions in Ugandan communities due to high levels of stress, stigma, anxiety, and depression mainly due to social isolation and the impact of economic losses as a result of the COVID-19 pandemic [10, 11].

Community Health workers (CHWs) are recognized globally for their role in the improvement of primary health care service delivery [12]. CHWs have been instrumental in supporting preventive, promotive, and curative health service delivery among communities for many years. CHWs have been involved in the management of previous epidemics such as: the Ebola outbreak of 2014–2016 in Liberia, Guinea, and Sierra Leone [13–15]; the HIV/AIDS epidemic in sub-Saharan Africa [16]; the Cholera outbreak in Haiti and Yemen [17]; and the Zika virus outbreak in the Americas [18–20].

CHWs have been at the forefront of the COVID-19 pandemic globally, particularly in low resource settings [21]. CHWs around the world have been involved in the COVID-19 response by carrying out various roles such as screening, information dissemination, awareness creation, supporting vaccine registration processes, mobilizing the community for vaccination, and supporting treatment [22]. They also played a critical role in maintaining access to essential services during the pandemic, notably community-based management of childhood illness. CHWs in Uganda, locally referred to as village health teams (VHTs), have been involved in creating awareness on COVID-19 and its preventive measures [23], and contact tracing [24], while others have supported vaccination [25, 26]. During the pandemic, the Government of Uganda through the Ministry of Health (MOH) has relied on CHWs to support contact tracing, community case management, as well as health promotion in addition to their routine roles [23, 25, 26].

A recent project implemented as part of the partnership between Makerere University School of Public Health and Nottingham Trent University (UK), in collaboration with Wakiso District Local Government, and MOH trained 766 CHWs in Wakiso district, Uganda on epidemic and pandemic preparedness and response including COVID-19. This training was conducted due to the vital role played by CHWs in supporting health systems at the grassroots, particularly during epidemics and pandemics [22, 27]. The 2-day participatory training was facilitated by health practitioners from local health facilities that supervise CHWs. The main topics in the training were: introduction to epidemics and pandemics; community engagement; contact tracing; risk communication; frontline protection; as well as community awareness and sensitization. The training also had a session on prevention and control of COVID-19 in the community. In addition, group discussions, sharing past experiences from common epidemics in Uganda, pre- and post-training assessments, and a certificate awarding ceremony were held. The pre- and post-training assessment carried out demonstrated improved knowledge and skills among the CHWs. In the pre-training assessment, 7.5% of the CHWs said they had adequate knowledge on epidemics and pandemics compared to 92.8% in the post-training assessment. Pre-training assessment results also showed that CHWs who said they had knowledge and skills required in community engagement, risk communication, and contact tracing were 65.7%, 55.6%, and 30.0% respectively compared to 99.4%, 99.4%, and 97.9% in the post-training assessment respectively. Furthermore, 65.9% of the CHWs said that they knew how to protect themselves while responding to epidemics and pandemics during the pre-training assessment compared to 100% in the post-training assessment. Although this post-training assessment was conducted immediately after the training, the long-term effects of the project on the CHWs and communities they serve was not established hence necessitating an impact evaluation.

Our evaluation is informed by a results chain that establishes causal logic from the initiation of the project, beginning with resources available, to the end, looking at long term goals [28]. The results chain sets out a logical, plausible outline of how a sequence of inputs, activities, and outputs for which a project is directly responsible, as well as interacts with behaviour to establish pathways through which impacts are achieved. A results chain maps the inputs, activities, outputs and outcomes of the project. In our project results chain, the inputs were the financial, human and other resources needed for implementation including funding, project staff, community mobilisers, trainers, and training materials. The activities in our results chain were the actions taken to convert inputs to outputs specifically mobilising and training CHWs on epidemic and pandemic preparedness and response.

The outputs in our results chain were the products resulting from converting inputs into tangible deliverables notably improved knowledge and skills of CHWs on epidemics and pandemics (which was assessed using the pre- and post-training assessment). The outcomes in our results chain (which are assessed in this evaluation) include short- and long-term effects of the intervention. This impact evaluation aimed to generate evidence on the outcomes of the project that could be used by local stakeholders including MOH, Wakiso District Local Government, and other implementing partners in preparedness and response to future epidemics and pandemics in the country.

## Methodology

### Study area and setting

The study was conducted in Wakiso district located in the central region of Uganda which neighbours Kampala, the country’s capital city. Wakiso is the most populated district in Uganda with an estimated population size of 2,915,200 inhabitants. The average household size is 4.7 and the main occupation being subsistence farming [29]. The district has a mix of urban and rural areas, with half of its population living in urban settings. The high population growth rate in the district poses a threat to the existing scarce resources and a challenge to service provision [30]. As such, CHWs are a great contribution to the human resource for health in the area.

The 6 local administrative units in Wakiso district which were involved in the study were Entebbe municipality, Bussi sub county, as well as Kajjansi, Katabi, Kasanje, and Kyengera town councils. These units had been involved in the project that trained the CHWs as part of the partnership between Nottingham Trent University and Makerere University School of Public Health. According to the last National Population Census (2014) [29], the population of the target local authorities is estimated as follows: Entebbe municipality (70,219), Bussi sub-county (15,827), Kasanje town council (29,008), Kajjansi town council (92,916), Katabi town council (105,669), and Kyengera town council (195,531) hence a total of 509,170. Given that this census was conducted over 7 years ago, and the population growth rate of Wakiso district of 4.1% [31], the current total population of the target area is likely to be over 1,000,000.

### Study design and participants

This was a qualitative evaluation that used three data collection methods: in-depth interviews (IDIs), focus group discussions (FGDs), and key informant interviews (KIIs). This evaluation was carried out in 2021, one year after the CHWs were trained on epidemics and pandemics. A total of 31 IDIs among trained CHWs, 15 FGDs among community members served by CHWs, and 11 KIIs among community health stakeholders including Wakiso district health team members, local leaders, and health practitioners who supervise CHWs were conducted.

### Sampling and data collection

CHWs who participated in the IDIs were identified purposively by their parish coordinators in the different administrative units. Six CHWs were selected in each administrative unit considering their gender, age, and disability. The IDIs were conducted among 14 men and 17 females, among whom 03 CHWs were persons with disability. For the FGDs, 2 rural and 1 urban parish / ward were randomly selected from the 6 administrative units, from which one village was randomly selected to be involved in the study. Community members were involved in the study as the trained CHWs were expected to have interacted with them following the training as beneficiaries of their services. The community members who participated in the study were purposively selected by local council I leaders of the respective villages. The leaders worked closely with community mobilizers to select the FGD participants. The FGDs included 9 mixed groups (both male and female), 3 male groups, and 3 female groups, each comprising of 8 to 9 participants. The KIIs were conducted among various stakeholders including the Wakiso district health team members, local leaders, and health practitioners who supervise CHWs. The number of IDIs, FGDs, and KIIs was sufficient to reach data saturation.

All data collection tools were developed by the researchers in line with the training conducted in the pilot project (see supplementary file). The FGDs and IDIs were conducted in *Luganda*, the most common local language spoken in Wakiso district, while the KIIs were conducted in either English or *Luganda* depending on the participant. All the interviews and discussions audio recorded. All FGDs were facilitated by two research assistants (RAs) who were trained and oriented by the principal investigator to ensure they were well versed with the tools before data collection. In addition, the RAs had prior experience in qualitative research. One RA moderated the FGDs, while the other did the audio recording and took notes. These RAs were not part of the team that facilitated the training during the earlier project. Data collection lasted approximately one hour for the FGDs, 40–60 min for the IDIs, and between 35 and 45 min for the KIIs. Data collection was conducted in observance of the COVID-19 prevention guidelines including the use of hand sanitizers, wearing of face masks, and observance of physical distancing.

All the data collection tools used were informed by vast literature on evaluation [32–37], as well as the materials used during the training on epidemics and pandemics. The tools focused on information regarding epidemics and pandemics particularly for COVID-19 following the training. The main aspects assessed by the tools were: enhancement of skills of CHWs; confidence of CHWs during their work; any changes (short, medium, and long term) in the performance of duties of CHWs; changes in the trends of morbidity and mortality in the community including for COVID-19; changes in the community’s structure, processes and behaviours as a result of interaction with CHWs; and any unintended consequences as a result of the training.

### Data management and analysis

The audio recordings of the FGDs, IDIs, and KIIs were transcribed verbatim and proofread by the RAs to ensure that they were accurate. For the interviews which were conducted in *Luganda*, the audio recordings were transcribed and translated to English, and the translation was verified by another researcher. The transcripts were then imported into NVivo (2020) software where data analysis was done by 3 researchers. The thematic analysis approach was used to guide the analysis process. The researchers read the transcripts several times to familiarize themselves with the data. Thereafter, words or related phrases from the transcripts were grouped to form codes. Coding was done by the 3 researchers in NVivo to group words, set of words, or phrases that were related. Thereafter, the codes were discussed by the entire research team and revised accordingly. The codes were then defined, and several quotes representing different codes were highlighted to develop a code book. Related codes were then grouped to form sub-themes, and related sub-themes were grouped together to form themes. The themes that were generated from the analysis informed the findings from the study.

## Results

Findings from the study are presented under four themes: improved knowledge and skills on managing epidemics and pandemics; enhanced attitudes towards managing epidemics and pandemics (CHWs showed dedication to their work and enhanced confidence); improved health practices in the community; and enhanced performance in managing epidemics and pandemics by CHWs.

### Improved knowledge and skills in managing epidemics and pandemics

#### Improved knowledge on epidemics and pandemics

From the IDIs, CHWs were able to distinguish between epidemics and pandemics, as well as gave relevant examples of each. The CHWs defined pandemics as disease outbreaks that spread across countries or international borders, while epidemics as outbreaks that occur within a specific community or region.

*“Epidemics are diseases that we learnt about that if they break out in a certain area*,* they don’t spread to many places*,* and there are pandemic diseases that break out and spread over a large area or all over the world like COVID-19.”*** Male participant 03**,** Bussi sub county**,** IDI**.

The CHWs identified several diseases that could either be epidemics or pandemics following the training such as COVID-19, cholera, Ebola, Marburg, Anthrax, and Rift Valley Fever. The CHWs were also able to identify the different signs and symptoms of some these diseases such as: bleeding from body openings, diarrhoea, vomiting, high body temperatures, and general body weakness for Ebola; diarrhoea, stomach ache, headache, vomiting, and blood stains in stool for cholera; and flu, cough, runny nose, sore throat, fever, loss of smell, loss of appetite, dryness of the throat, and difficulty in breathing for COVID-19.

*“For Ebola*,* someone has a lot of diarrhoea sometimes with blood stains*,* a lot of vomiting*,* high body temperature*,* and general body weakness*,* while someone with COVID-19 experiences body weakness*,* flu*,* and a sore throat.”*** Female participant 04**,** Kyengera Town Council**,** IDI**.

#### Improvement of skills

CHWs stated that they had learnt many skills from the training such as communication, treatment of illnesses, and report writing. Many CHWs reported that they gained skills in carrying out community awareness and sensitization on COVID-19 such as health educating people on how to protect themselves through wearing masks, handwashing, and social distancing. Some CHWs also stated that the treatment skills gained helped a lot in managing COVID-19 patients including provision of home-based care. Many CHWs highlighted that they were able to write reports on different health related incidents, as well as communicate effectively with their colleagues on any health-related issue in their communities.

*“It is no longer hard for me to support someone who for example has COVID-19 because I know the right steps. I was taught and I have the skills to treat them and they recover. Before the training*,* I would even fear to go near a COVID-19 patient*,* but now I know how to handle them*,* and how to advise the caretakers for those doing homebased care.”*** Female participant 02**,** Katabi Town Council**,** IDI**.

Some CHWs also stated that previously, they did not know the proper procedure of washing their hands or wearing gloves but after the training, they had learnt such techniques. Some highlighted that they had increased their handwashing frequency since the training. They also identified that wearing gloves while handling a suspected case can help reduce the risk of transmitting communicable diseases.

*“We got many skills. I even didn’t know how to wash my hands well but now*,* I know how to wash my hands and how many times they should be washed. I also know how to wear gloves and I know that someone can get the disease if they don’t know how to wear the gloves properly.”*** Male participant 03**,** Bussi sub county**,** IDI**.

### Enhanced attitudes towards managing epidemics and pandemics

#### Dedication to work

Throughout the FGDs, community members reported that the attitudes of CHWs towards their work had improved. According to the FGD participants, they had watched CHWs carry out their duties in the community with passion. They also stated that CHWs worked very hard, and invested their time to take health services closer to their populations. Some community members acknowledged that at times CHW service came with personal costs such as time and finances for example during community mobilization and engagement, yet many CHWs were always willing to pay the cost to ensure the wellbeing of their community.

*“They love their work a lot and I have seen many of them sacrifice their time to health educate the community on diseases including COVID-19. For someone to leave their home and walk the whole village*,* it shows that such a person loves their work.”*** Female participant 02**,** Katabi Town Council**,** Mixed FGD**.

#### Enhanced confidence

A common view among the IDI participants was that after the training, the CHWs had become more confident regarding managing epidemics and pandemics. Many CHWs alluded to the fact that before the training, they did not know much about COVID-19 or how to manage it in their community hence were not actively involved in engaging on health issues. In addition, other CHWs mentioned that they also had fear because they thought that once they acquired COVID-19, the end result was death, so they stayed away from any possible chances of contracting it. However, it was reported that during the training, they acquired knowledge about the disease, how it is spread, how to manage and prevent it, as well as how to protect themselves as frontline health workers. This gave them the confidence to mobilise and health educate the rest of the community, while speaking from an informed point of view.

*“I become more confident because when COVID-19 had just started to spread*,* we were not confident and I used to fear. However*,* we were trained on the ways I can use to prevent contracting the disease*,* I became more confident and courageous because I got to know that if I practice what they had taught me*,* I would prevent the spread of COVID-19.”*** Female participant 04**,** Kasanje Town Council**,** IDI**.

KII and FGD participants also agreed that the confidence of the CHWs to work was amplified by the training on epidemics and pandemics. Some KIIs also highlighted that this improvement was not only seen within their communities but also in comparison to CHWs in other areas that had not received such similar training.

*“The most outstanding difference is that these CHWs who were trained are more confident because they have the knowledge and certificates from the training. They are very confident because*,* in a meeting we recently had*,* you could tell the difference as*,* you would find the trained CHW could explain health issues on COVID-19 correctly*,* and even elaborated with examples when compared to other CHWs who never received the training. For them* [non-trained CHWs], *they were not participating actively in the discussions.”*** Female health worker participant 01**,** Kyengera Town Council**,** KII**.

### Improved health practices in the community

From the FGDs, community members attributed improved health practices in their households to the work of CHWs. According to the FGD participants, they had learnt many health promotion actions from CHWs which were effective in controlling and preventing the spread of COVID-19. These practices included wearing masks properly, having more than one reusable mask to allow changing in case it got dirty, proper handwashing at home, avoidance of crowded areas, and observing social distance. Other community members stated that the habits they acquired were also transferrable to other family members such as their children. Among the IDI participants, many CHWs also reported improved hand hygiene at the community and household levels, as well as increased uptake of COVID-19 vaccination following the training.

*“Yes*,* the change that was noticed is that when I taught people in the community*,* they got to know that COVID-19 can spread not only to adults but also young children*,* and also that COVID-19 kills. During COVID-19 vaccination in my village*,* children whose parents had kept them from earlier vaccination*,* accepted to have them vaccinated.”*** Female participant 03**,** Kasanje Town Council**,** IDI**.

Many of the CHWs highlighted that they were initially fearful about getting vaccinated against COVID-19 before the training. This was because they did not have much information about the disease besides what was communicated over the radio by MOH, and the misinformation on social media. However, after the training, many of the CHWs had a change in perspective, as they became aware of the vital role of vaccination in curbing further spread of the virus. This finding was also echoed among key informants who stated that after the training, most CHWs immediately went for vaccination.

*“After the training*,* the CHWs knew the importance of vaccination in controlling the pandemic. Actually*,* we vaccinated all CHWs in our area and they all received their second dose! What is remaining is the booster dose and all that is attributed to the training. The training greatly enhanced knowledge and practices and such a change would not have happened without the activity.”*** Female Health worker participant 02**,** Kasanje Town Council**,** KII**.

### Enhanced performance in managing epidemics and pandemics

Community members reported that CHWs carried out community mobilization through door-to-door visits and talks on community radio as part of the COVID-19 response. The CHWs also mentioned that they increased the frequency of the number of household visits they made during the COVID-19 pandemic after the training. According to them, the training created this increase as these home visits were conducted to ensure that people in their communities were kept safe from COVID-19. During the visits, the CHWs reported to have encouraged people to go for COVID-19 testing, and advocated for vaccination of vulnerable groups such as the elderly, especially those with underlying illnesses such as diabetes, TB, and HIV.

*We also have TB and HIV patients*,* so those are the ones we were constantly visiting because they were at a high risk of getting serious effects if they got sick*,* with COVID-19. We would tell them to go and get vaccinated and when they returned*,* we would regularly visit them to see that they are well. We also did this for the elderly.”*** Male participant 05**,** Kajjansi Town Council**,** IDI**.

The same view was also expressed by the FGD participants as they stated that CHWs played a crucial role in managing the COVID-19 pandemic. Many of them highlighted that CHWs encouraged them to wash their hands; ensured that each household had a handwashing facility; gave out face masks to some people and encouraged wearing them; encouraged social distancing; advised people to avoid crowded places; and provided home-based treatment for those who were infected. In addition, the community members stated that after the training, the CHWs also taught them how to make hand washing facilities in their homes during the COVID-19 pandemic.

*“I once suffered from COVID-19 but the CHWs could visit all the time and they encouraged me to isolate myself from the rest of the people*,* avoid moving a lot*,* and having water and soap for handwashing when I don’t have a sanitizer. They used to visit us to check how we were doing while sick and also told us not to go for burials which attracted large crowds.”*** Female participant 08**,** Kyengera Town Council**,** Female FGD**.

Furthermore, key informants applauded the CHWs for supporting patients with chronic diseases during the COVID-19 pandemic. Some key stakeholders mentioned that CHWs helped to get medication for people suffering from chronic diseases such as HIV/AIDs from health facilities. This was because these people did not have access to transport means to the facilities. Other key informants also appreciated the CHWs for being discreet as they delivered the health services without disclosing the health status of their community members.

*“There were HIV patients who did not have drugs*,* but the CHWs would walk to the health facility and pick the drugs for them. As you know*,* HIV patients like their privacy hence the CHWs would reach out to them*,* counsel and help them. So that training was really helpful.”*** Male Local Council leader participant 01**,** Katabi Town Council**,** KII**.

Beyond pandemics and epidemics, community members stated that CHWs offered them usual routine health services such as treating malaria, diarrhoea, and pneumonia among children under five years, counselling, health education, distribution of mosquito nets, conducting referrals to health facilities, carrying out household visits, as well as community mobilization for vaccination, and family planning after the training. By offering such services, community members did not have to make such trips to the health centers themselves during the pandemic.

*“The CHWs have offered us services because they brought us nets*,* because of that*,* we did not have to go to hospitals where COVID-19 patients were. So*,* we remained in our homes. Imagine if we had someone with malaria and that person was to go to a hospital managing COVID-19 cases for treatment frequently. The chances of our household members acquiring COVID-19 would have been high*.” ** Male participant 4**,** Katabi Town Council**,** Mixed FGD**.

## Discussion

CHWs, if well supported can improve health outcomes within their communities during epidemics and pandemics [13, 38–41]. Our study shows that the CHWs trained during the project demonstrated improved knowledge, skills, and enhanced attitudes toward managing epidemics and pandemics. This led to better performance of CHWs when handling incidences of epidemics and pandemics in their community particularly for COVID-19. The recognition of CHWs as the first line of Uganda’s health system in the community necessitates governments and implementing partners to invest in this cadre, as this evaluation shows that enhancing the capacity of CHWs can contribute to preparedness and response to outbreaks.

Our results showed that CHWs exhibited improved knowledge and skills on epidemics and pandemics as shown in previous studies where evaluations were carried out among this cadre [42, 43]. It is important to note that this evaluation was carried out one year after the training. The CHWs from the study correctly distinguished between epidemics and pandemics, and identified examples and signs of diseases associated with them. This finding is consistent with the project post-assessment results that found out that over 90% of the CHWs reported increased knowledge of epidemics and pandemics. Participants from our study reported improvement in skills such as communication, treatment of illnesses, and report writing following the training. As shown by previous studies, good communication skills are of great importance when handling misinformation in the community [40, 44, 45]. In addition, good report writing skills allowed for contact tracing and general surveillance of suspected cases in past pandemics [15]. Enhanced knowledge and skills of CHWs is therefore likely to contribute to improved performance during pandemics and epidemics.

Community members in this study noted that the CHWs were more dedicated and confident in their work after the training. Indeed, CHWs themselves reported being more confident with using frontline protection, and demystifying COVID-19 miscommunication especially explaining concerns and myths around vaccination. Previous studies have also reported increased confidence among CHWs to educate communities after various capacity building initiatives [39, 46]. Indeed, acquiring the right information on health issues through timely training increases the motivation and capacity of CHWs to advise on health promotion activities [45, 47]. The positive attitude of CHWs towards managing epidemics and pandemics established in our study could have been contributed to by the use of demonstrations on hand washing and wearing of gloves as protective gear; presentation of COVID-19 vaccination cards by training facilitators; and the ample time taken to explain the history of COVID-19 and how vaccination works, as part of the training.

Results from the study showed that CHWs continued to provide routine services such as Integrated Community Case Management (iCCM) of childhood illnesses, distribution of nets, and referral of cases during the COVID-19 pandemic. This finding is similar to a study carried out in Bangladesh that found that some CHWs maintained their regular routine service levels, despite the lockdown that hindered other frontline workers and health assistants from carrying out community-based services due to movement restrictions [45]. However, this finding differs from earlier studies where CHWs were not able to work effectively during the pandemic due to movement restrictions, fear of acquiring the disease, and lack of access to personal protective equipment [48–50]. Access to essential routine health services often declines during pandemics and epidemics [13, 51]. However, this evaluation showed that CHWs can support to bridge that gap if trained.

Particularly to COVID-19, the findings from our study showed increased community mobilization for uptake of vaccination; contact tracing; door-to-door and community radio health education; and household visits, as shown by previous systematic reviews [39, 40] and studies in Bangladesh [52], Uganda [48, 53] and USA [43]. Furthermore, CHWs in our study reported to prioritise COVID-19 health services for the elderly and those with chronic illnesses such as HIV/AIDS. Enhanced access to drugs through CHWs among HIV/AIDs patients during the COVID-19 pandemic has also been documented elsewhere in Uganda [54].

Health practices such as vaccination, hand hygiene, and wearing of face masks in the community were enhanced in our study following the training. This can be attributed to the fact that CHWs can empower communities to respond and act to health concerns as shown in previous studies among underserved communities in the USA [43, 46, 55], Nigeria [56] and West Africa [57]. CHWs usually stay in the communities they serve and therefore are well positioned and trusted messengers to address any misinformation, fear, and stigma if knowledgeable [22, 58, 59]. In addition, many of the CHWs were vaccinated after the training hence being exemplary in their communities. This underscores the importance of having a well-trained community health workforce.

Overall, our training enhanced the capacity of CHWs in the prevention and control of epidemics and pandemics, especially COVID-19. The lessons learnt from this project can be applied to other emerging and re-merging diseases in Uganda such as Cholera and Ebola. We believe several reasons were responsible for the success of this project:

- The rigorous 2-day training focusing on several topics such as introduction to epidemics and pandemics; community engagement; contact tracing; risk communication; frontline protection; and community awareness and sensitization.
- The use of demonstrations, group discussions, and illustrations as part of the training.
- The timing of the training allowed facilitators to refer to the ongoing COVID-19 pandemic.
- The ongoing COVID-19 outbreak allowed the CHWs to immediately put the acquired knowledge and skills into practice.
- Dedicated support supervision and mentorship of CHWs by their supervisors who also doubled as their training facilitators.
- Working within the already existing government health structures by the CHWs. Indeed, training was held among already existing CHWs in the community who are part of the health system.

Interpretation of the findings should consider that the CHW training was carried out before the second wave of the COVID-19 pandemic in Uganda. The training therefore utilised the earlier experiences and lessons from the first wave such as the disruption of medical supplies and distrust of CHWs by the community with support from the MOH. As the evaluation was carried out 1 year after the training, there was the potential for recall bias on some of the aspects assessed. In addition, some of the improvements in knowledge and practices among the CHWs may be attributed to other initiatives beyond the project. Nevertheless, the use of different qualitative data collection methods (focus group discussions, key informants, and in-depth interviews) enhanced rigour of the study, as well as enabled triangulation of findings. To the best of our knowledge, this study is among the first to evaluate the knowledge, skills and performance of CHWs in the prevention of epidemics and pandemics in Uganda following a capacity building initiative.

## Conclusion

Training of CHWs on epidemics and pandemics enhanced their knowledge, skills, attitudes, practices, and performance while managing outbreaks particularly COVID-19. The training also enhanced dedication and confidence of CHWs to perform their roles in the community during the outbreak. Capacity building of CHWs should be considered as a crucial activity to support the health system during epidemics and pandemics.

### Electronic supplementary material

Below is the link to the electronic supplementary material.

Supplementary Material 1

## Data Availability

The datasets used and/or analysed during the current study are available from the corresponding author on reasonable request.

## Abbreviations

AIDS: Acquired immunodeficiency syndrome
CHWs: Community Health Workers
FGDs: Focus Group Discussions
HIV: Human Immunodeficiency Virus
IDIs: In-depth Interviews
KIIs: Key Informant Interviews
MOH: Ministry of Health, Uganda
TB: Tuberculosis
VHTs: Village Health Teams
