# Supplementary material for: Enhancing the capacity of community health workers in prevention and control of epidemics and pandemics in Wakiso district, Uganda: evaluation of a pilot project
Source: BMC Prim Care. 2024 Jul 17;25:260. doi: 10.1186/s12875-024-02522-1 (PMC11253445; doi:10.1186/s12875-024-02522-1)
Supplement: Supplementary file 1 — Supplementary Material 1 [file 12875_2024_2522_MOESM1_ESM.docx]

## In-depth Interview guide for Village Health Teams – English

**Enhancing the capacity of Community Health Workers on epidemic and pandemic preparedness and response in Wakiso district, Uganda: impact evaluation and dissemination**

Thank you for accepting to take part in this evaluation. First of all, I would like to ask you some background questions on your role as a VHT. Thereafter, I would like to talk to you about the epidemic and pandemic preparedness and response training (including COVID-19) that was organised by Makerere University last year.

**NOW SWITCH ON THE RECORDER**

1. **Background questions**
2. Initials of the VHT
3. What is the name of the health facility you are attached to (Katabi HC II, Nagula HC II, Kigungu HC III, Bussi HC III, Kajjansi IV, Kasanje HC III, Entebbe Regional Referral Hospital, Kyengera HC III, Nsangi HC III, Nakitokolo HC II, Kasenge HC II, Zzinga HC II)
4. Which sub-county / town council do you serve? (Entebbe municipality, Bussi sub-county, Kasanje town council, Kajjansi town council, Katabi town council and Kyengera Town councils)
5. What is the parish: ………….. and village ………………..?
6. What is your age (in complete years)?
7. Gender (Female or male)
8. What is your highest educational qualification? *(None, Primary (P1 to P7), Secondary (O level – S1 to S4, A level S5 to S6, Certificate, Diploma, Bachelors degree, Masters degree, etc.)*
9. What is your VHT role? (iCCM or non-iCCM)
10. How long have you worked in this role?
11. **Evaluation questions**
12. What can you tell me about the epidemic and pandemic preparedness and response training (including for COVID-19) that was organised by Makerere University last year? What do you remember best about that 2-day training?  ***Probe****: Epidemics and pandemics - Ebola, Marburg, Rift Valley, Measles, Cholera, Crimean Congo haemorrhagic, Anthrax, Meningitis, COVID-19, etc. Can you identify any signs, symptoms and preventive measures for these diseases? What is the difference between epidemics and pandemics? What was the overall significance of the epidemic and pandemic preparedness and response training? What do you understand by the following terminologies: community engagement, contact tracing, risk communication, community awareness and sensitization in relation to control and prevention of pandemics and epidemics?*
13. Did you learn any new skills / have any skills been enhanced as a result of the epidemic and pandemic preparedness and response training organised by Makerere University last year? If yes, please mention the new skills learnt / enhanced? ***Probe:*** *skills on community engagement, contact tracing, risk communication, frontline line protection, case identification, counselling, referrals, community awareness and sensitization. How have these skills being used in their day-to-day work as a VHT? Can they give an example of how these skills have been used in their work in the community – probe regarding number home visits, referrals, health education sessions, follow up visits that have been made, etc. in relation to pandemics and epidemics particularly for COVID-19?*
14. Following the training on epidemic and pandemic preparedness and response held last year, did you become more confident / less confident in aspects of your work in the community as a VHT? ***Probe:*** *For example, did they learn something that they have used or that they would use during future situations as may be required?*
15. Did the training on epidemic and pandemic preparedness and response improve your role as a VHT in your community? If so, provide more details. ***Probe:*** *Did the training improve their household visiting / sensitization / community mobilization / contact tracing / community surveillance / home management of patients in relation to epidemics and pandemics particularly for COVID-19?*
16. Did performing your duties together with other VHTs regarding epidemic and pandemic preparedness and response in the community contribute to improved health as well as reduction in morbidity and mortality particularly for COVID-19? If so, kindly provide details on this contribution. ***Probe:*** *what activities did they participate in following the training? Explore in relation to community engagement, risk communication, sensitization, contact tracing, etc. Are there any changes in their community that they can attribute to this training?*
17. Have you been involved in creating awareness on epidemic and pandemic preparedness and response (including for COVID-19) in your community following the training organized by Makerere University held last year? If so, kindly provide details about this involvement. ***Probe:*** *How much time have they put aside for this activity? How often do they carry out this activity in a week? What motivates them to continue creating awareness on epidemic and pandemic preparedness and response?*
18. Have you set any specific goals regarding epidemic and pandemic preparedness and response (including for COVID-19) in your community following the training organized by Makerere University held last year? ***Probe:*** *If yes, please tell me why and what goals / targets have you set? Has it been feasible for them to meet these set goals / targets?*
19. How easy or difficult is it to remember the key points from the epidemic and pandemic preparedness and response training organized by Makerere University held last year? ***Probe****: What are some of the things that have enabled them to remember these key points - facilitators, group work, experience, notebooks, supervision, follow-up, etc?*
20. Did your working environment facilitate the performance of your role as a VHT in epidemic and pandemic preparedness and response (including for COVID-19)? If so, kindly provide more details. What barriers / facilitators exist within your environment? ***Probe:*** *are there enough resources such as availability of PPE and other supplies, vaccinations, referral forms, hand washing facilities, supervisors, following of recommended guidelines, any cultural perceptions and practices related to disease prevention and control, etc. What are the some of the barriers preventing you from performing your work?*
21. Do you ever receive feedback on your performance as a VHT on epidemic and pandemic preparedness and response (including for COVID-19) from your supervisor / fellow VHTs / VHT coordinator / implementing partners or community? ***Probe:*** *Who provides this feedback? Why or why not? Provide details of this feedback.*
22. Is there any other information you may want to provide regarding the recent training on epidemic and pandemic preparedness and response (including for COVID-19) held last year and how it could have supported your role as VHT?

**Thank you for your time.**

**Key Informant Interview guide (English)**

**Enhancing the capacity of Community Health Workers on epidemic and pandemic preparedness and response in Wakiso district, Uganda: impact evaluation and dissemination**

Thank you for accepting to take part in this study. First of all, I would like to ask you some background questions on your role as a stakeholder in community health. Thereafter, I would like to talk to you about the epidemic and pandemic preparedness and response training (including COVID-19) that was organised by Makerere University last year.

**NOW SWITCH ON THE RECORDER**

1. **Background questions**
2. Initials of the stakeholder
3. What is the name of the health facility you are attached to (if applicable) (Katabi HC II, Nagula HC II, Kigungu HC III, Bussi HC III, Kajjansi IV, Kasanje HC III, Entebbe Regional Referral Hospital, Kyengera HC III, Nsangi HC III, Nakitokolo HC II, Kasenge HC II, Zzinga HC II)
4. What is your age (in complete years)?
5. Gender (female or male)
6. What is your highest educational qualification? *(None, Primary (P1 to P7), Secondary (O level – S1 to S4, A level S5 to S6, Certificate, Diploma, Bachelors degree, Masters degree, etc.)*
7. What is your role in relation to VHTs? (VHT supervisor, district health team member, or local leader)
8. How long have you worked in this role?
9. **Evaluation questions**
10. Last year, Makerere University organised a training on epidemic and pandemic preparedness and response including COVID-19 among VHTs in Wakiso district. Do you know anything this training? ***Probe***: *Was the training modality appropriate? Was the programme executed as intended? Why or why not?* *Can you identify possible reasons to explain your views?*
11. Are there any changes (short, medium and long term) in the performance of duties of VHTs that can be attributed to the recent training on epidemic and pandemic preparedness and response (including COVID-19) organised by Makerere University last year? Please mention any changes. ***Probe:*** *changes in attitudes / practices / beliefs, knowledge / confidence / behaviour, etc. of VHTs?*
12. Are there any changes in the community’s structure, processes and behaviours as a result of interaction with VHTs who participated in the training on epidemic and pandemic preparedness and response (including COVID-19)? ***Probe:*** *Changes in hand washing practices, cultural customs, latrine use, health seeking behaviours, observing COVID-19 SOPs, etc.*
13. Have there been any activities that VHTs have participated in that may have contributed to changes in the trends of morbidity and mortality including for COVID-19 following the recent training on epidemic and pandemic preparedness and response*?* ***Probe:*** *If yes, please elaborate these activities of the VHTs? If not, why are there no possible changes? Are there health indicators (especially for COVID-19 such as vaccination rates) that you can relate to these changes? How could the VHT activities have contributed to any observed changes?*
14. Are there any unintended consequences as a result of the recent training on epidemic and pandemic preparedness and response (including COVID-19) among VHTs? ***Probe:*** *If yes, can you mention these consequences? Consequences can be: experiences of those who have been affected by the programme including VHTs, health workers, community members, implementing partners; change in community structures; more support from the district, Ministry of Health, and other implementing partners; mentorship of VHTs from other areas; recognition of VHTs by various stakeholders; enhanced collaboration between VHTs, community members and local leaders; etc.*
15. What is your view on such 2-day training workshops to enhance the capacity of VHTs? ***Probe:*** *Are they adequate? Is more time needed? Are they beneficial? Are they effective in terms of reach? Are the impacts like to be sustainable? Explain why or why not?*
16. Would you recommend scale-up of such a training to other VHTs in Wakiso district and other districts in the country? ***Probe:*** *Why or why not?*
17. Is there any other information you may want to provide regarding the recent training on epidemic and pandemic preparedness and response including for COVID-19 held last year and how it could have benefitted VHTs, community members, and the broader health system?

**Thank you for your time.**

## Appendix 11: Focus Group Discussion guide – community members (English)

**Enhancing the capacity of Community Health Workers on epidemic and pandemic preparedness and response in Wakiso district, Uganda: impact evaluation and dissemination**

Thank you for accepting to take part in this study. First of all, I would like to ask you some background questions about yourself. Thereafter, I would like to talk to you about a training organised for village health teams (VHTs) by Makerere University on epidemic and pandemic preparedness and response training (including COVID-19) to your community.

**NOW SWITCH ON THE RECORDER**

1. **Background questions**
2. Initials of the community member
3. Which sub-county / town council do you stay? (Entebbe municipality, Bussi sub-county, Kasanje town council, Kajjansi town council, Katabi town council and Kyengera town council)
4. What is the parish: ………….. and village ………………..?
5. What is your age (in complete years)?
6. Gender (female or male)
7. What is your highest educational qualification? *(None, Primary (P1 to P7), Secondary (O level – S1 to S4, A level S5 to S6, Certificate, Diploma, Bachelors degree, Masters degree, etc.)*
8. How long have you lived in this area?

**B. Evaluation questions**

1. Do you know VHTs in your area, their homes, the services they provide, and their availability? ***Probe:*** *Explain in detail why or why not? Which services do they provide? How often do you seek their services in regard to disease prevention, curative services and health promotion? Are you able to access their services in a timely manner?*
2. Are there any changes (short / medium term – less than 1 year and long term – 1 year and above) in your community, or behaviour that can be attributed to your interactions with VHTs? Can you think of a scenario when you adopted a healthy behaviour in your home or community related to epidemics / pandemics particularly COVID-19 as a result of being in contact with VHTs? If possible, describe a scenario in relation to epidemic and pandemic preparedness and response including for COVID-19. ***Probe:*** *Scenarios can include moments in which you were actively involved in community health activities such as environmental cleaning, personal hygiene and sanitation, COVID-19 prevention and management, etc.*
3. To what degree do you consider the roles played by VHTs as important and valuable to the health and wellbeing of the community? ***Probe****: Health education and sensitization, treatment of childhood diseases, case identification, contact tracing, follow up of patients and referrals critical for the health and wellbeing of the community?* *Have you ever saved money that would otherwise be used for health expenditure due to the services of a VHT - please share your experience?*
4. Are you satisfied with and confident in the services VHTs are offering to this community related to epidemics and pandemics particularly COVID-19? Are the services of VHTs able to meet your local needs? Services include referrals, risk communication, contact tracing, community awareness and sensitization. ***Probe:*** *On a scale of 1 to 10, how satisfied and confident are you with these services. Explain in detail why or why not?*
5. VHTs in your area received a specific training on epidemics and pandemics including COVID-19 last year. How have the VHTs in your area supported prevention and control of diseases in the recent past, particularly for COVID-19? ***Probe*:** *Have you consulted or has a VHT visited you to educate you about epidemic and pandemic preparedness and response (including COVID-19)? What was the role of VHTs in observance of COVID-19 SOPs, vaccination, home based management of COVID-19 patients, surveillance, etc.*
6. In your own opinion, do you believe that VHTs are willing to do / doing their best regarding community health especially in correspondence with the health needs of this community especially during the COVID-19 pandemic? ***Probe:*** *Why or why not? How does this relate to their performance during the COVID-19 pandemic?*
7. Is there any other information you may want to provide regarding the work of VHTs including during the ongoing COVID-19 pandemic?

**Thank you for your time.**
